# Acute monocular nasal hemianopia following a mild traumatic brain injury: A case report

**DOI:** 10.1097/MD.0000000000021352

**Published:** 2020-07-24

**Authors:** Hsin-Le Lin, Ju-Chuan Yen

**Affiliations:** a Graduate Institute of Biomedical Informatics, College of Medical Science and Technology, Taipei Medical University, Taipei, Taiwan; b Department of Ophthalmology, Ren-Ai Branch, Taipei City Hospital, Taipei, Taiwan; c Department of Education and Research, Taipei City Hospital, Taipei, Taiwan; d University of Taipei, Taipei, Taiwan.

**Keywords:** hemianopia, indirect traumatic optic neuropathy, optic nerve, traumatic brain injury

## Abstract

**Introduction:**

Monocular hemianopia is a visual field defect with an uncommon pattern. The etiology of monocular temporal hemianopia has been well-evaluated and has been suggested to result from an optic nerve or chiasmal lesion. However, the etiology of monocular nasal hemianopia remains unclear.

**Patient concerns:**

Here, we present the case of a 41-year-old male who was punched on the head with fists during a fight and then suffered from painless blurred vision in the left eye after mild traumatic brain injury. An ophthalmic examination revealed a conjunctival chemosis, periorbital hematoma, and a relative afferent pupillary defect in the left eye. Automated perimetry indicated there was a left side nasal hemianopia along the vertical meridian.

**Diagnosis:**

Examination of the fundus showed there was a normal appearing retina and disc bilaterally. Fluorescein angiography revealed no delayed filling of the vessels. Computed tomography and magnetic resonance imaging showed unremarkable findings of the visual pathways, orbit, and brain. A diagnosis of left traumatic optic neuropathy was made.

**Interventions:**

Systemic steroid pulse therapy (1 gram of intravenous methylprednisolone per day) was given to the patient for 3 days.

**Outcomes:**

An ophthalmologic examination after treatment indicated there was no obvious improvement in the relative afferent pupillary defect, best corrected visual acuity, and color sense. A second set of automated perimetry results showedno changes after 3 months.

**Conclusion:**

Monocular nasal hemianopia caused by traumatic optic neuropathy is uncommon. In this case, monocular nasal hemianopia was likely due to ischemic changes from impairment of the prechiasmal arterial anastomotic network or indirect injury to the lateral prechiasmal nerve fiber.

## 1 Introduction

Monocular hemianopia is a visual field defect with an uncommon pattern. Temporal hemianopia cases have been reported and are considered to be the result of an optic nerve or chiasmal lesion.^[1]^ However, monocular nasal hemianopia along the vertical meridian is extremely rare and its etiology is still unknown. Here, we report a case of a monocular nasal hemianopia associated with indirect traumatic optic neuropathy (TON) following mild traumatic brain injury.

## 2 Case presentation

A 41-year-old male was seen at the emergency department after being involved in a fight with 2 men. He reported being grabbed around the neck and then was punched on the head with fists during the fight. He immediately developed periorbital bruises in the left eye and left side painless blurred vision.

**Figure 1:**
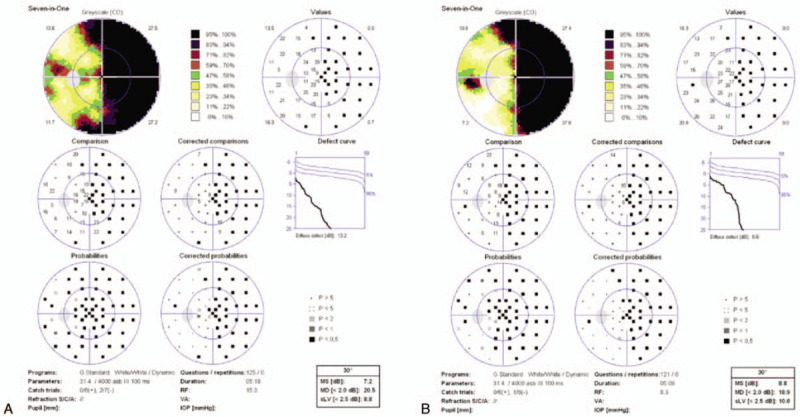
Visual field. 1A. Automated perimetry recorded from the left eye at initial presentation demonstrate nasal hemianopia. 1B. Repeated automated perimetry recorded 3 months after systemic steroid pulse therapy.

**Figure 2:**
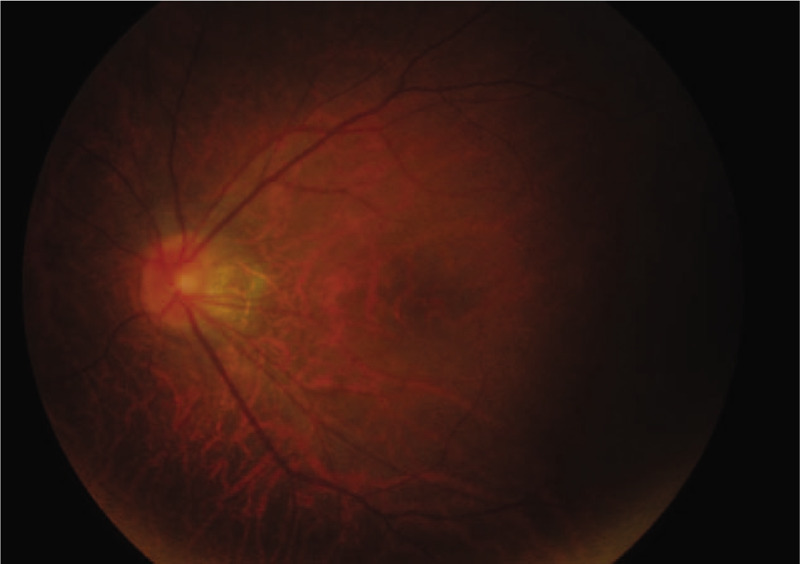
Examination of the left eye fundus demonstrated a normal appearing disc and retina.

In the emergency department, the patient's vital signs remained stable and the Glasgow coma scale score was greater than 14. He developed dizziness, post-traumatic amnesia, and a headache that did not last more than 24 hours. There were minor abrasions on the extremities and some ecchymosis around the neck. On ophthalmic examination, his best-corrected visual acuity was 20/100 in the left eye and 20/20 in the right eye. A left eye examination revealed a periorbital hematoma, conjunctival chemosis, and a relative afferent pupillary defect. Farnsworth-Munsell 100 hue results revealed a left side dyschromatopsia. The patient also reported that Snellen chart letters corresponding to the nasal field were obscured in his left eye. Automated perimetry and a confrontation test indicated left side nasal hemianopia along the vertical meridian (Fig. 1). The visual field defect showed a clear margin along the central vertical meridian with a mean deviation of 20.5 dB. The right eye visual field was shown to be normal. Other cranial nerve functions were normal. Intraocular pressure was within the normal limit. Extraocular muscle movement remained free and full in all directions. Examination of the fundus demonstrated a normal appearing retina and disc bilaterally (Fig. 2).

In consideration of traumatic brain injury and cerebral microemboli, further evaluation was carried out, including carotid Doppler ultrasonography, computed tomography angiography, ophthalmic artery Doppler ultrasonography. The results revealed no evidence of atherosclerosis nor emboli. Fluorescein angiography revealed no delayed filling of vessels. A magnetic resonance imaging (MRI) scan showed unremarkable findings in the brain, orbit, and visual pathways. A diagnosis of left TON was made.

The patient received systemic steroid pulse therapy (1 gram of intravenous methylprednisolone per day) for 3 days. An ophthalmologic examination performed after treatment showed no obvious improvement in best corrected visual acuity, color sense and the relative afferent pupillary defect. Several weeks later the temporal part of the optic disc became pale. Repeated automated perimetry results were unchanged after 3 months.

Informed patient consent was obtained from the patient for publication of this case report.

## 3 Discussion

Monocular hemianopia is a visual field defect with an uncommon pattern. The etiology of monocular temporal hemianopia has been well-evaluated and has been suggested to result from an optic nerve or chiasmal lesion.^[1]^ However, the etiology of monocular nasal hemianopia remains unclear.

Several case reports had been published in the literature documenting unilateral nasal visual field defects. The underlying causes included a giant carotid-ophthalmic artery aneurysm, a temporal arachnoid cyst, sphenoid wing meningioma, and ethmoid sinus mucocele.^[2–5]^ In reviewing these reports, all space-occupying lesions caused a mass compressive effect to the lateral part of prechiasmal optic nerve, which served the nasal visual field. It is believed that a monocular nasal hemianopia results in a compressive lesion to the ipsilateral prechiasmal optic nerve.^[2]^

Manor et al classified intracranial causes of nasal visual field loss into 5 categories:

(1) vascular,
(2) tumoral,
(3) internal hydrocephalus with distended third ventricle,
(4) inflammatory, and
(5) traumatic.^[6]^

The possible pathogenesis of a nasal visual field defect might be circulation impairment of the prechiasmal arterial plexus.

Rahman et al reported a case of unilateral nasal hemianopia resulting from a posterior subcapsular cataract.^[7]^ In this case report, the patient developed sudden onset of blurry vision affecting her right nasal visual field. Slit lamp examination revealed a marked central posterior subcapsular lens opacity. A cataract extraction was performed after establishing the absence of neurologic and cardiovascular lesions. A repeat visual field showed complete improvement of the visual field defect postoperatively.

In review of published case reports, none of these cases were caused by trauma. To the best of our knowledge, this is the first case of monocular nasal hemianopia caused by minor head injury induced TON. TON was defined as visual loss from direct or indirect trauma causing acute injury to the optic nerve. Causes of TON can vary from high-velocity eye trauma, such as a motor vehicle accident, bike accident, and falling debris, to trivial causes such as following an endoscopic sinus surgery.^[8]^ Damage to the optic nerve leads to axonal dysfunction, causing corresponding visual field defects before changes occur in the fundoscopic appearance. The characteristics of visual field defects could be an index for estimating TON and provide a reference for the clinical diagnosis. The distribution of visual field defects in TON varies. Generalized constriction and depression, altitudinal defects, hemianopia, ring scotoma, arcuate scotoma, as well as central and paracentral scotomas can present, which depends on different intensities and directions of contusion force damage to the optic nerve fiber.^[9]^

In cases of indirect TON caused by trivial head trauma, often no obvious ophthalmoscopic signs are seen initially. There are several typical hallmarks that can be used to diagnose indirect TON:

(1) visual acuity loss,
(2) variable visual field defects,
(3) color vision loss, and
(4) an afferent pupillary defect.^[10]^

Indirect TON causes not only neuronal and axonal injury, but also choriocapillaries perfusion impairment. Chan et al analyzed 19 veterans who suffered from indirect TON in mild chronic traumatic brain injury.^[10]^ They found 3 major patterns of optical coherence tomography changes in these patients:

(1) temporal peripapillary retinal nerve fiber layer thinning,
(2) superior side macular ganglion cell layer thinning greater than on the temporal side, and
(3) subfoveal choroidal thinning.

This study confirmed the optic neurovascular injuries from a trivial head injury.

In our case, we assumed that a minor blunt head trauma caused shearing forces that were transmitted to the lateral part of the prechiasmal optic nerve fibers or to the vascular supply of the nerve. However, it is unknown why the traumatic force predominantly damage the nondecussating lateral fibers rather than causing diffuse stretching of the nerve. The persistence of the monocular nasal hemianopia might have resulted from irreversible circulatory impairment in the prechiasmal arterial anastomotic network, causing irreversible ischemic changes.^[6]^

Mild traumatic brain injury is defined as traumatic head injury with conscious loss less than 30 minutes, a Glasgow coma scale score of 13 or more, and amnesia less than 24 hours.^[10]^ In some uncomplicated brain concussion cases, computed tomography scans or MRI may not detect brain structural damage. However, clinicians should keep in mind that limited structural axonal injury and permanent loss of axonal function might be present without radio-image evidence. In our case, it is also a possibility that a lesion that was too small for imaging on MRI may have been present. Patients with no obvious fundoscopic and radio-image findings or those who compensate for monocular visual field loss with binocular viewing may have a delay in diagnosis and treatment. In addition, the incidence of monocular hemianopia may be under-estimated.

## 4 Conclusion

Monocular nasal hemianopia due to TON is uncommon. TON can result from a trivial trauma without obvious eyeball or optic nerve injuries based on the gross appearance or under ophthalmoscopy. TON can lead to any form of visual field defect. Monocular nasal hemianopia was likely due to indirect injury to the lateral prechiasmal nerve fiber or ischemic changes from impairment of the prechiasmal arterial anastomotic network.

## Acknowledgments

We would like to express our thanks to all colleagues and students who contributed to this study. We thank the editor, series editor and reviewers for their constructive comments.

## Author contributions

**Conceptualization:** Hsin-Le Lin.

**Data curation:** Hsin-Le Lin.

**Investigation:** Hsin-Le Lin.

**Supervision:** Ju-Chuan Yen.

**Writing – original draft:** Hsin-Le Lin.

**Writing – review and editing:** Ju-Chuan Yen.
